# Efficacy of platelet-rich plasma and plasma for symptomatic treatment of knee osteoarthritis: a double-blinded placebo-controlled randomized clinical trial

**DOI:** 10.1186/s12891-021-04706-7

**Published:** 2021-09-24

**Authors:** Murillo Dório, Rosa Maria Rodrigues Pereira, Alexandre Galeno Branco Luz, Leticia Alle Deveza, Ricardo Manoel de Oliveira, Ricardo Fuller

**Affiliations:** 1 Rheumatology Division, Hospital das Clínicas da Faculdade de Medicina da Universidade de São Paulo, São Paulo, Brazil; 2 RDO Diagnósticos Médicos, São Paulo, Brazil; 3 Rheumatology Department, Royal North Shore Hospital and Institute of Bone and Joint Research, Kolling Institute, University of Sydney, Sydney, New South Wales Australia

**Keywords:** Knee osteoarthritis, Plasma, Platelet-rich plasma, PRP

## Abstract

**Background:**

Platelet-rich plasma (PRP) has a still conflicting efficacy for knee osteoarthritis (KOA) and might be a minimally invasive and safe treatment alternative. The potential benefit of only plasma (non-enriched) has never been investigated. Our aim was to evaluate the efficacy of intra-articular platelet-rich plasma (PRP) and plasma to improve pain and function in participants with KOA over 24 weeks.

**Methods:**

Randomized, double-blind, placebo-controlled trial with 3 groups (n = 62): PRP (n = 20), plasma (n = 21) and saline (n = 21). Two ultrasound-guided knee injections were performed with a 2-week interval. The primary outcome was visual analog scale 0-10 cm (VAS) for overall pain at week 24, with intermediate assessments at weeks 6 and 12. Main secondary outcomes were: KOOS, OMERACT-OARSI criteria and TUGT.

**Results:**

At baseline, 92% of participants were female, with a mean age of 65 years, mean BMI of 28.0 Kg/m^2^and mean VAS pain of 6.2 cm. Change in pain from baseline at week 24 were -2.9 (SD 2.5), -2.4 (SD 2.5) and -3.5 cm (SD 3.3) for PRP, plasma and saline, respectively (p intergroup = 0.499). There were no differences between the three groups at weeks 6 and 12. Similarly, there were no differences between groups regarding secondary outcomes. The PRP group showed higher frequency of adverse events (65% versus 24% and 33% for plasma and saline, respectively, p = 0.02), mostly mild transitory increase in pain.

**Conclusions:**

PRP and plasma were not superior to placebo for pain and function improvement in KOA over 24 weeks. The PRP group had a higher frequency of mild transitory increase in pain.

**Trial registration:**

ClinicalTrials.gov, NCT03138317, 03/05/2017.

**Supplementary Information:**

The online version contains supplementary material available at 10.1186/s12891-021-04706-7.

## Background

Knee osteoarthritis (KOA) is estimated to affect over 10% of the population worldwide [1] with a lifetime risk of 45% [2]. Current guidelines recommend both non-drug (such as exercise) and drug therapies (such as anti-inflammatory agents) [3, 4]. However, these therapies generally have short-term benefits and effect sizes are only small to moderate [5, 6]. Furthermore, use of drugs is restricted in patients with comorbidities due to the risk of adverse events [3]. Intra-articular glucocorticoids are generally recommended only for short-term pain relief given that benefits are limited to few weeks [3, 7], and a recent study suggested that repeated injections are associated with increased cartilage loss [8]. Hyaluronic acid (HA) use is controversial, and guidelines provide conditional recommendations [3, 4]. Having failed these options, knee arthroplasty is usually an effective definitive treatment, but it is expensive and there is the risk of medical and post-surgical complications [9]. Thus, identifying alternative efficacious and safe treatments for KOA is important.

Biological treatments have been recently studied for treatment of knee OA such as platelet-rich plasma (PRP) [10], an autologous blood product that contains an elevated concentration of platelets. The release of growth factors and other molecules, including platelet-derived growth factor, transforming growth factor-β, type I insulin-like growth factor and vascular endothelial growth factor is supposed to be related to its efficacy [11]. Several clinical trials have shown that PRP may be promising for KOA treatment [12–14]. However, most of them are conflicting regarding the methods and present many limitations that hinder an adequate analysis of their results, with risk of bias [13, 15]. Heterogeneity in the preparation and injection methods used by published studies is a limitation for determining optimal PRP protocols [12, 14]. Furthermore, the majority of trials has the HA as comparator, which is itself controversial [12]. A few trials compared PRP to placebo so far, with results showing significantly greater improvements in symptoms over saline at 6 and 12 months [16–20]. However, these studies suffered from major methodological flaws including lack of adequate blinding, suggesting that the benefits may have been overestimated [12]. Thus, further studies comparing PRP to placebo are still necessary.

Human plasma is composed of water, several proteins and electrolytes, coagulation factors and immunoglobulins. As it is the medium in which the concentrated platelets of the PRP are diluted, the study of its possible action for improving the outcomes in KOA is important. The advantages of PRP or plasma for treatment for KOA would be: i) it is relatively easy to use as preparation is rapid and it is minimally invasive; ii) it is a relative low-cost treatment, considering the use of existing structure and equipment in public health services; and iii) it is likely to be safe as it is an autologous product and previous studies reported only minor and transient adverse effects [12]. Therefore, the aim of this study was to evaluate the efficacy of PRP and plasma for improving pain and function in participants with KOA over 24 weeks.

## Methods

### Study design

Clinical trial of superiority, randomized, double-blind, placebo-controlled, parallel, with 3 groups with a 1:1:1 allocation ratio. We have compared two active treatment groups, one receiving PRP and the other receiving plasma only, with a control group that received saline. Two ultrasound-guided joint injections were performed with a 2-week interval and outcomes were evaluated at week 24, with intermediate assessments at weeks 6 and 12. We have followed the Osteoarthritis Research Society International (OARSI) recommendations for KOA trials in the conduct of this study [21]. The study protocol was registered at ClinicalTrials.gov, NCT03138317, with first date of registration 03/05/2017.

### Sample

The study was carried out at the Hospital das Clinicas Rheumatology Department of the University of São Paulo (HC-FMUSP), a tertiary hospital in São Paulo, Brazil. The sample consisted of participants diagnosed with KOA in rheumatology outpatient clinics and external participants advertised by word of mouth from the community and screened by telephone and then personally at the clinic. All participants were instructed about the study and signed an informed consent form.

The following criteria were used for inclusion: 1) men and women aged 45 to 80 years; 2) fulfill criteria for KOA of the American College of Rheumatology [22]; 3) radiographic grade 2 or 3 scored by the Kellgren and Lawrence (KL) [23] in at least one knee; 4) pain from 3 to 8 cm in the visual analogue scale 0-10 cm (VAS) in at least one knee in the last week. Knee x-rays obtained within 6 months before allocation were accepted. The knee selected for treatment was the one reported with higher pain score as reported by the participant.

Exclusion criteria were: 1) use of analgesics, non-steroidal anti-inflammatory drugs, myorelaxants and systemic glucocorticoids within one week to allocation; 2) use of slow acting drugs for OA (such as chondroitin, glucosamine, diacerein) started within 8 weeks to allocation. For participants using these drugs for longer than 8 weeks, they could be maintained until the end of the study; 3) corticosteroids or HA intra-articular injection in the index knee within 6 months to allocation; 4) intra-articular injection of any drug in any other joint within 1 month to allocation; 5) introduction of any medical or physical intervention for the locomotor system within the last 3 months (exercise, acupuncture, cane, orthotics etc.); 6) KL 4 in any of the knees; 7) body-mass index (BMI) ≥ 35 kg/m^2^; 8) fibromyalgia and inflammatory arthropathies such as rheumatoid arthritis, connective tissue diseases, microcrystalline arthropathies, spondyloarthropathies and infectious arthropathies; 9) symptomatic OA of hips or feet; 10) previous surgery in the index knee; 11) difference in length of lower limbs > 1 cm; 12) skin lesion on index knee surface; 13) any blood dyscrasia (including thrombocytopenia) or use of anticoagulants; 14) other diseases: severe depression, non-controlled diabetes, decompensated cardiovascular disease, infection, immunosuppression (methotrexate up to 10 mg/week was allowed), systemic infectious disease, symptomatic lower limb vascular disease, neurological diseases, cancer or any other conditions believed to interfere with results; 15) any sick leave or similar due to KOA.

### Procedures

The screening and follow-up visits were realized at Hospital das Clinicas and all baseline clinical assessment and intervention procedures were undertaken in a private laboratory. The VAS for overall pain questionnaire was applied at screening and baseline assessments to ensure that participants continued to fulfil inclusion criteria and reduce the influence of fluctuation in pain in the screening-baseline assessment period, as recently raised by some authors [24–26]. Baseline clinical assessment consisted of self-reported questionnaires, physical examination and physical tests, and collection of blood for full blood count. Participants were also underwent to ultrasonographic assessment [27, 28] of the index knee at baseline before treatment administration (Additional file 1). A single radiologist with more than 20 years of experience in ultrasonography of the musculoskeletal system and interventional procedures (AGBL) performed all ultrasound evaluations and guided injections and a single investigator (MD) performed all clinical assessments.

The knee injections were ultrasound-guided, performed using the superolateral approach, 2 cm above and laterally to the superolateral angle of the patella using a 22G needle, with administration of local anesthetic (2% lidocaine without vasoconstrictor) subcutaneously for analgesia only. Using the same needle, participants received one of the three treatments (PRP, plasma or saline) according to allocation group. A bandage (Blood Stop®) was applied with orientation to be removed the day after the procedure. Participants were instructed to avoid exercises within 48 hours after the procedure.

Throughout the study, participants were allowed to use paracetamol in case of pain, at maximum dosage of 3g/day, and to avoid the use of other medications, mainly anti-inflammatory and dipyrone (metamizole).

### Randomization, allocation and blinding

A randomization list was generated in blocks of 3 by a person not involved in the study, using the version 2015a of the MATLAB Program® and the allocation generation ratio was 1:1:1. For allocation concealment, correspondent letters for each treatment group were placed into sequentially numbered opaque envelopes according to the randomization list and then were sealed. These procedures were also done by a person not involved in the study. Each participant who has consented to enter the study was destined an envelope in the sequence. Allocation of participants occurred from March 2017 to October 2017 and the assessments were completed in March 2018.

All participants were submitted to blood collection to maintain blinding. The envelopes were opened sequentially by the laboratory pharmacist who prepared treatments, not involved in the study. After the preparation of the treatment injections by the pharmacist, the syringes were covered completely (including the needle base) with a non-transparent white adhesive label only with the participant's identification and delivered to the radiologist who performed the guided injections. All researchers involved in the study including the statistician who performed the analysis were blinded. Inadvertent unblinding of the radiologist/injector (AGBL) has not been assessed although he was not involved in the assessment of outcomes or data analysis. All participants were blinded. The revelation of the treatment groups occurred only after statistical analysis and production of the main results.

### Preparation of PRP and plasma

**Fig. 1:**
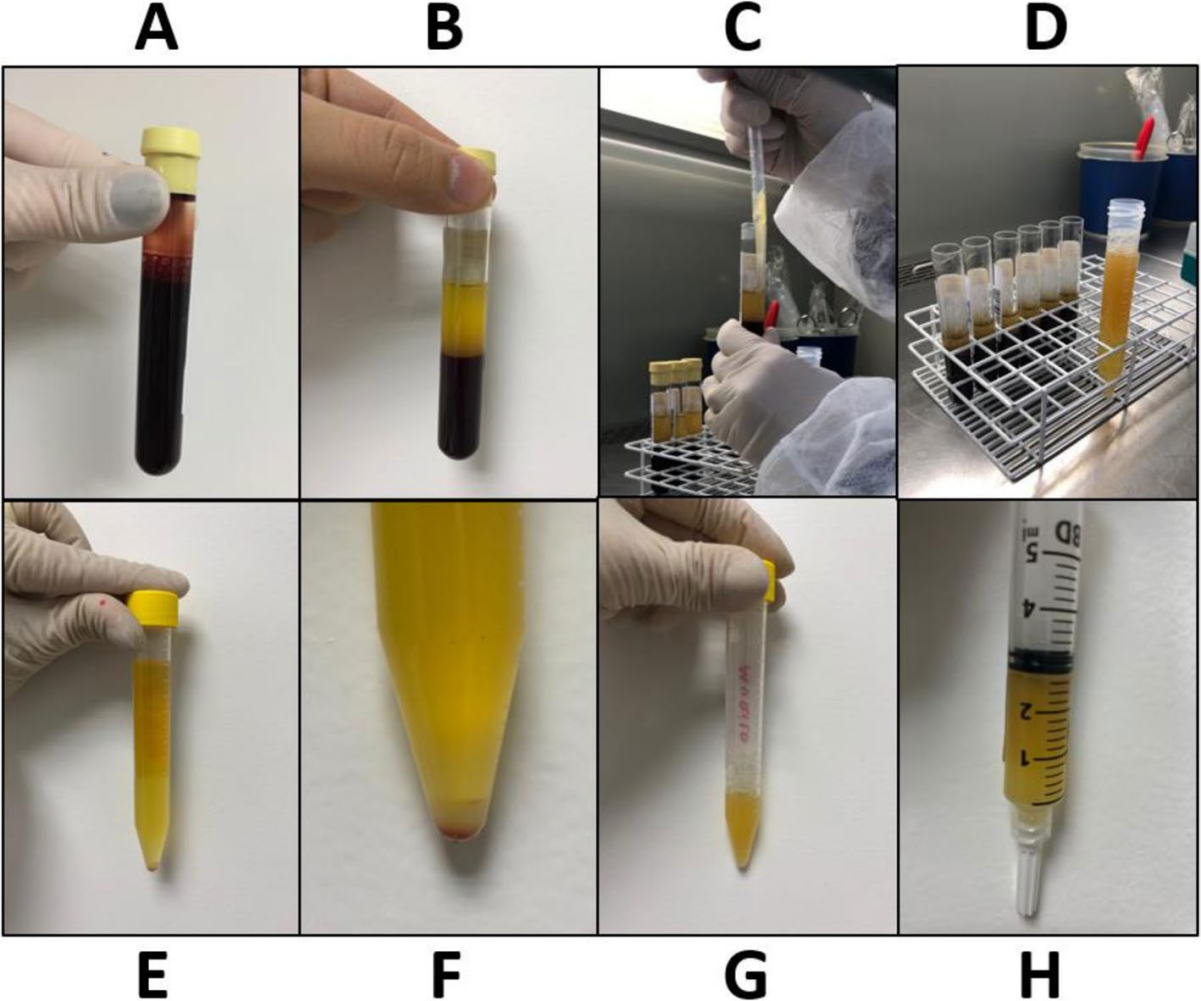
Preparation of platelet-rich plasma (PRP) and plasma: **A** – blood collected; **B** – after 1^st^ centrifugation, separation of erythrocytes on the bottom, an intermediate thin layer known as buffy coat and a superior layer of plasma; **C** – separation of plasma from all tubes; **D** – separated plasma in a centrifuge tube (Falcon®); **E** – after 2^nd^ centrifugation, separation of a pellet of platelets on the bottom and plasma (without platelets); **F** – magnification of the pellet of platelets; **G** – plasma withdrawal (for use as treatment injections) and redilution of the pellet of platelets for obtention of a PRP with approximately 1 x 10^6^ platelets/mm^3^; **H** – final PRP for treatment injections

Participants were submitted to collection of 40 ml of autologous blood by the antecubital vein in vacuum tubes (BD Vacutainer®) of 10 ml containing as anticoagulant the sodium citrate solution at 3.8% (Fig. 1). Tubes were submitted to double centrifugation (Cel LS3 Plus® centrifuge). The first spin was performed at 1500 rpm (395 g) lasting 12 minutes. After 10 minutes of resting, an inferior layer consisting of erythrocytes, an intermediate layer with buffy coat and a superior layer of plasma were obtained. This plasma was submitted to a second spin at 2300 rpm (930 g) lasting 10 minutes [29] with separation of a pellet of platelets from plasma (without platelets). At this stage, plasma was obtained for use as treatment injections; and redilution of the pellet of platelets was undertaken to obtain a PRP with approximately 1 x 10^6^ platelets/mm^3^ (approximately 3 times the basal platelet count) also for use as treatment injections [29]. No activation was added to PRP.

Pre-centrifugation platelet count in peripheral blood was used to estimate the final volume of PRP to maintain the concentration of 1 x 10^6^ platelets/mm^3^ in the injections. The final volume of injected PRP ranged from 1.4 to 5 ml for each participant. The volume of plasma and saline were also calculated according to the count of platelets in peripheral blood for each participant to maintain the blinding of the radiologist who did the injections (1.4 to 5 ml). Platelets, leukocytes and erythrocytes were counted after centrifugations in some samples of both PRP and plasma to confirm their expected concentration. Both PRP and plasma had approximately zero leucocytes and erythrocytes. The plasma used for treatment also had approximately zero platelets.

All procedures were performed in biological-protector hoods (Veco Bioprotector®), according to the recommendations of the *Cell Medicine Society* [30]. The treatments were prepared in the laboratory RDO by a single pharmacist, who works in the laboratory, not involved in the study.

### Outcomes

Primary outcome was change in VAS 0-10 cm for overall pain in the index knee at week 24. Secondary outcomes were: 1) VAS 0-10 cm for pain at rest; 2) VAS 0-10 cm for pain at movement; 3) VAS 0-10 cm for physician global assessment (PhGA); 4) participant’s global assessment (PGA) for improvement, 0-100%; 5) Western Ontario McMaster Universities Osteoarthritis Index (WOMAC), 5-point Likert 0-4 for each question [31, 32]; 6) Knee Injury and Osteoarthritis Outcome Score (KOOS), 5-point Likert 0-4 for each question [33, 34]; 7) Likert scale for improvement: no improvement, mild improvement, moderate improvement, good improvement, excellent improvement; 8) OMERACT-OARSI criteria [35] defined as A) improvement in pain or function ≥ 50% and absolute improvement ≥ 20 or B) improvement in at least 2 of the following 3 criteria: i) pain ≥ 20% and absolute improvement ≥ 10, ii) function ≥ 20% and absolute improvement ≥ 10; iii) PGA ≥ 20% and absolute improvement ≥ 10. For these criteria, pain was assessed by VAS for overall pain and function was assessed by WOMAC function subscale; 9) timed up and go test (TUGT) [36]; 10) analgesic consumption, assessed by a self-reported diary.

### Statistical analysis

Sample size was calculated for the primary outcome at week 24. A 2 cm reduction in VAS for overall pain was considered as the minimum difference to be detected between the groups with a standard deviation (SD) of 1.5, based on the study by Patel et al [16]. An alpha of 5% and a power of study (1 - beta) of 80% were used. Fifteen participants were required in each of the three groups. To allow a loss of follow-up of 20%, the minimum number of participants in each group was 18. The calculation was performed by the Action Stat Pro® software.

Mean and SD were obtained for continuous variables and distribution was presented for categorical variables. To compare groups at baseline, the Chi-square or Fisher's exact tests were used for the categorical variables and ANOVA or Kruskal-Wallis for continuous variables. For data collected at 6, 12 and 24 weeks, the ANOVA test with repeated measures for continuous variables. Non-parametric tests were used for evaluation of Likert scale (Friedman test for paired data) and OMERACT-OARSI criteria (Cochran's Q test for paired data). We performed a post-hoc subanalysis to investigate potential predictors of improvement with PRP or plasma treatment. The analyses were conducted using the software SPSS version 20. A statistical significance of 5% was considered for all tests and we did not adjust for multiplicity. Missing data were treated with multiple imputation.

## Results

**Fig. 2:**
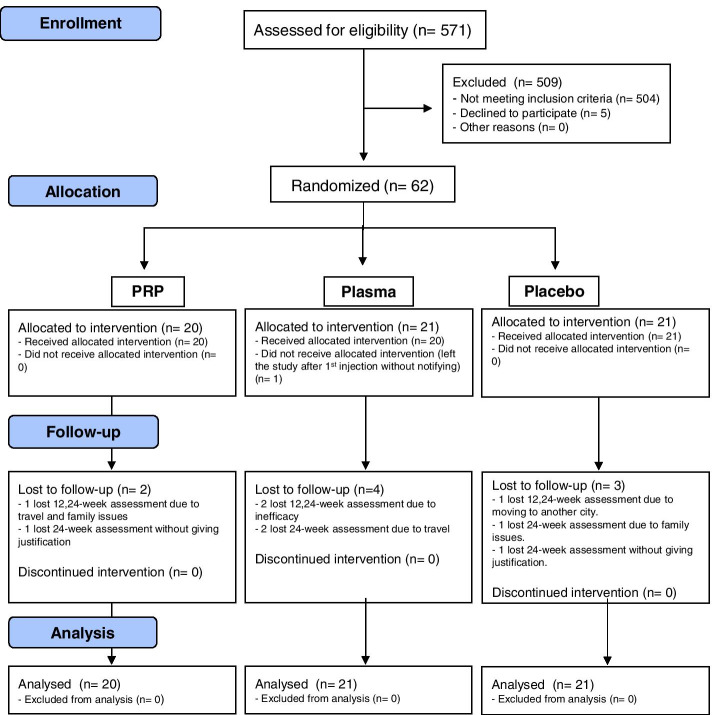
CONSORT diagram

Five hundred seventy-one participants were screened and most excluded, mainly due to an end-stage KOA (KL 4) or a severe systemic disease (rheumatic or not) given that our OA clinic is at a tertiary hospital. Sixty-two participants were allocated to treatment groups (Fig. 2): 57 (92%) were female, with mean age of 65 years and mean BMI of 28.0 Kg/m^2^. All groups were homogeneous at baseline, except for the KOOS domain for sport and recreation in which the plasma group presented higher score in relation to the PRP group, and for the presence of synovial hypertrophy in ultrasonography in which the PRP group presented lower degrees for the gray scale score in relation to the other groups (Table 1).

**Table 1:** Characteristics at baseline

Variables	PRP (n = 20)	Plasma (n = 21)	Placebo (n = 21)
Age in years, mean (SD)	66.4 ± 5.6	66.1 ± 7.5	62.5 ± 8.1
Sex, n (%)			
Male	1 (5%)	2 (10%)	2 (10%)
Female	19 (95%)	19 (90%)	19 (90%)
BMI, mean (SD)	28.3 ± 4.1	28 ± 3.1	27.6 ± 3.8
BMI – category, n (%)			
< 25	6 (30%)	4 (19%)	4 (19%)
25-30	5 (25%)	11 (52%)	11 (52%)
30-35	9 (45%)	6 (29%)	6 (29%)
Knee, n (%)			
Left	9 (45%)	9 (43%)	11 (52%)
Right	11 (55%)	12 (57%)	10 (48%)
X-ray, n (%)			
KL2	13 (65%)	13 (62%)	14 (67%)
KL3	7 (35%)	8 (38%)	7 (33%)
Physical Activity, n (%)	0 (0%)	0 (0%)	1 (6%)
No	12 (60%)	9 (43%)	9 (43%)
Yes	8 (40%)	12 (57%)	12 (57%)
Comorbidities, n (%)			
No	4 (20%)	5 (24%)	3 (14%)
Yes	16 (80%)	16 (76%)	18 (86%)
Duration of symptoms in years, mean (SD)	8.4 ± 6.5	7 ± 8.1	7.1 ± 6.9
VAS 0-10 cm for pain, mean (SD)			
Overall	6.1 ± 1.6	5.9 ± 1.4	6.6 ± 1.4
At rest	2.5 ± 2.3	1.5 ± 1.9	1.9 ± 1.9
At movement	6.8 ± 2.1	6.8 ± 2.1	6.8 ± 2
PhGA 0-10 cm, mean (SD)	4.6 ± 1.9	4.6 ± 1.5	4.1 ± 1.7
WOMAC^a^, mean (SD)			
Pain 0-20	10.7 ± 3.2	9.2 ± 2.5	11 ± 3.1
Stiffness 0-8	4.4 ± 1.7	4 ± 1.4	4.3 ± 1.8
Function 0-68	37.9 ± 11.6	33.5 ± 11.2	37 ± 12
Total 0-96	52.9 ± 15.5	46.7 ± 14.3	52.3 ± 15.9
KOOS 0-100^b^, mean (SD)			
Symptoms	46.1 ± 21	56.1 ± 21.6	45.4 ± 15.9
Pain	42.9 ± 15.3	50.8 ± 17.5	40.7 ± 14.6
ADL	44.4 ± 15.3	51.8 ± 18.6	45.4 ± 16.2
Sport/Recreation	12.8 ± 10.9	29.8 ± 21	17.4 ± 12.2
QOL	18.1 ± 13.9	29.5 ± 16.4	25 ± 15.3
TUGT (s), mean (SD)	13.6 ± 2.8	13.8 ± 3.1	13.6 ± 3
US – synovitis, n (%)			
No	6 (30%)	2 (10%)	1 (5%)
GS1	8 (40%)	6 (29%)	2 (10%)
GS2	4 (20%)	5 (24%)	7 (33%)
GS3	2 (10%)	8 (38%)	11 (52%)
US - power doppler, n (%)			
No	16 (80%)	17 (81%)	18 (86%)
Grade 1	1 (5%)	3 (14%)	3 (14%)
Grade 2	3 (15%)	1 (5%)	0 (0%)
US – effusion, n (%)			
No	6 (30%)	2 (10%)	3 (14%)
0-2mm	2 (10%)	3 (14%)	1 (5%)
2-4mm	5 (25%)	6 (29%)	5 (24%)
4-6mm	4 (20%)	3 (14%)	6 (29%)
> 6mm	3 (15%)	7 (33%)	6 (29%)
US – cartilage^c^, n (%)			
1	0 (0%)	3 (14%)	1 (5%)
2A	13 (65%)	8 (38%)	13 (62%)
2B	3 (15%)	8 (38%)	4 (19%)
3	4 (20%)	2 (10%)	3 (14%)

^a^ higher number associated with worse evaluation

^b^ higher number associated with better evaluation

^c^ For cartilage classification, see the text

*Abbreviations*: *SD* standard deviation, *BMI* body mass index, in kg/m^2^, *KL* Kellgren and Lawrence classification for x-ray, *VAS* visual analogue scale, *PhGA* physician global assessment, *WOMAC* Western Ontario and McMaster Universities Index, *KOOS* Knee Injury and Osteoarthritis Outcome Score, *ADL* function in daily living, *QOL* quality of life, *TUGT* timed up and go test; US: ultrasonography; GS: gray scale

**Fig. 3:**
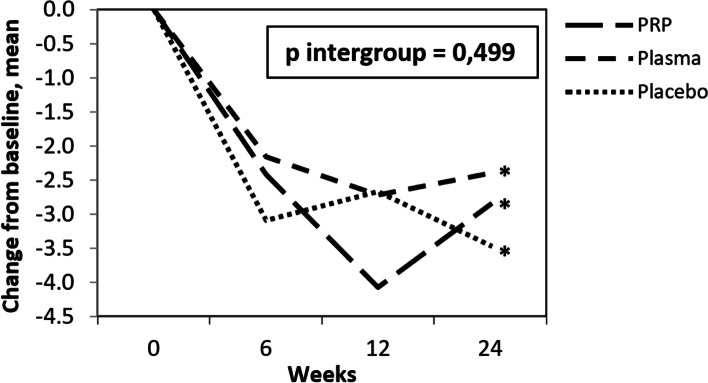
Visual analogue scale 0-10 cm for overall pain, change from baseline. *p intragroup < 0.001 (all 3 groups); p < 0.001 between baseline and weeks 6, 12 and 24; p > 0.05 between weeks 6, 12 and 24. 95% Confidence Intervals: PRP – week 6 (-3.6; -1.2), week 12 (-4.8; -3.4), week 24 (-4.0; -1.7); Plasma – week 6 (-3.1; -1.2), week 12 (-4.0; -1.4), week 24 (-3.5; -1.3); Placebo – week 6 (-4.2; -2.0), week 12 (-4.0; -1.3), week 24 (-4.9; -2.1).

The primary outcome, VAS for overall pain at 24 weeks, demonstrated significant improvement in the 3 groups, without statistical difference between them (Fig. 3, Table 2). The mean change from baseline for overall pain was -2.9 (SD 2.5) cm, -2.4 (SD 2.5) cm and -3.5 (SD 3.3) cm for PRP, plasma and saline groups, respectively (p intergroup = 0.499). Similarly, there were no statistical differences between the groups at weeks 6 and 12.

**Table 2:** Results

Outcomes	Groups	Baseline	Week 6	Week 12	Week 24	p-value intergroup*
VAS 0-10 cm for overall pain, mean (SD)	PRP	6.1 (1.6)	3.7 (2.4)	2.0 (1.4)	3.2 (2.5)	0.483
	Plasma	5.9 (1.4)	3.7 (2.5)	3.2 (2.8)	3.5 (2.4)	
	Placebo	6.6 (1.4)	3.5 (2.1)	3.9 (2.7)	3.1 (2.6)	
VAS for overall pain (change from baseline), mean (SD)	PRP	-	-2.4 (2.7)	-4.1 (1.6)	-2.9 (2.5)	0.499
	Plasma	-	-2.2 (2.3)	-2.7 (3.0)	-2.4 (2.5)	
	Placebo	-	-3.1 (2.5)	-2.7 (3.2)	-3.5 (3.3)	
VAS for overall pain, % of improvement, mean (SD)	PRP	-	-36.5 (39.7)	-67.0 (23.0)	-46.4 (41.3)	ns^a^
	Plasma	-	-36.2 (39.8)	-43.5 (55.6)	-38.0 (50.4)	
	Placebo	-	-43.4 (46.5)	-35.8 (52.5)	-46.5 (54.3)	
VAS 0-10 cm for pain at rest, mean (SD)	PRP	2.5 (2.3)	1.2 (1.6)	0.6 (0.8)	1.6 (2.2)	0.216
	Plasma	1.5 (1.9)	0.8 (1.1)	0.6 (0.7)	0.9 (0.9)	
	Placebo	1.9 (1.9)	1.6 (2.0)	1.0 (1.2)	1.4 (2.3)	
VAS 0-10 cm for pain at movement, mean (SD)	PRP	6.8 (2.1)	4.7 (2.8)	3.7 (2.3)	3.7 (2.8)	0.928
	Plasma	6.8 (2.1)	4.5 (3.1)	3.9 (3.1)	4.0 (2.7)	
	Placebo	6.8 (2.0)	4.2 (2.2)	3.9 (2.9)	3.5 (2.6)	
PhGA 0-10 cm, mean (SD)	PRP	4.6 (1.9)	3.3 (2.0)	2.3 (1.4)	3.0 (2.0)	0.634
	Plasma	4.6 (1.5)	3.1 (2.0)	2.9 (2.1)	3.1 (1.8)	
	Placebo	4.1 (1.7)	2.7 (1.8)	2.5 (1.5)	2.7 (2.0)	
PGA (0-100%), mean (SD)	PRP	-	48.0 (29.9)	67.3 (21.6)	56.3 (29.2)	0.639
	Plasma	-	59.0 (25.3)	63.2 (24.5)	67.7 (18.8)	
	Placebo	-	59.0 (27.0)	57.7 (31.1)	66.7 (25.4)	
Likert for global improvement^b^, %	PRP	-	0 – 10%	0 – 0%	0 – 5%	ns^c^
			1 – 15%	1 – 5%	1 – 20%	
			2 – 25%	2 – 20%	2 – 15%	
			3 – 45%	3 – 60%	3 – 35%	
			4 – 5%	4 – 15%	4 – 25%	
	Plasma	-	0 – 5%	0 – 0%	0 – 0%	
			1 – 10%	1 – 15%	1 – 5%	
			2 – 35%	2 – 20%	2 – 40%	
			3 – 45%	3 – 50%	3 – 35%	
			4 – 5%	4 – 15%	4 – 20%	
	Placebo	-	0 – 10%	0 – 14%	0 – 5%	
			1 – 10%	1 – 10%	1 – 14%	
			2 – 33%	2 – 19%	2 – 5%	
			3 – 38%	3 – 48%	3 – 67%	
			4 – 10%	4 – 10%	4 – 10%	
WOMAC Pain 0-20, mean (SD)	PRP	10.7 (3.2)	7.9 (3.7)	5.6 (2.6)	6.6 (3.5)	0.561
	Plasma	9.2 (2.5)	6.8 (3.6)	6.1 (3.9)	6.5 (3.6)	
	Placebo	11.0 (3.1)	7.4 (3.5)	7.1 (2.8)	6.2 (2.3)	
WOMAC stiffness 0-8, mean (SD)	PRP	4.4 (1.7)	2.8 (1.7)	2.1 (1.0)	2.7 (1.6)	0.713
	Plasma	4.0 (1.4)	2.9 (1.7)	2.6 (1.9)	2.5 (1.7)	
	Placebo	4.3 (1.8)	2.8 (1.6)	3.0 (1.5)	2.8 (1.5)	
WOMAC function 0-68, mean (SD)	PRP	37.9 (11.6)	25.8 (12.0)	21.2 (9.8)	23.5 (14.3)	0.847
	Plasma	33.5 (11.2)	25.4 (13.9)	20.9 (14.6)	24.2 (15.5)	
	Placebo	37.0 (12.0)	26.6 (12.5)	24.7 (10.0)	22.6 (11.0)	
WOMAC Total 0-96, mean (SD)	PRP	52.9 (15.5)	36.4 (16.7)	28.9 (12.6)	32.7 (18.9)	0.787
	Plasma	46.7 (14.3)	35.0 (18.5)	29.6 (19.9)	33.2 (20.3)	
	Placebo	52.3 (15.9)	36.9 (17.1)	34.9 (13.5)	31.6 (14.4)	
KOOS Symptoms 0-100, mean (SD)	PRP	46.1 (21.0)	67.0 (19.0)	71.4 (13.2)	63.9 (21.2)	0.442
	Plasma	56.1 (21.6)	66.3 (18.1)	69.4 (20.0)	66.0 (23.6)	
	Placebo	45.4 (15.9)	63.6 (15.8)	59.9 (17.1)	65.3 (17.4)	
KOOS Pain 0-100, mean (SD)	PRP	42.9 (15.3)	63.9 (18.7)	67.6 (12.5)	62.4 (20.1)	0.434
	Plasma	40.8 (17.5)	61.9 (19.3)	67.5 (21.9)	66.1 (21.0)	
	Placebo	40.7 (14.6)	58.7 (13.9)	59.4 (16.2)	66.1 (16.7)	
KOOS ADL 0-100, mean (SD)	PRP	44.4 (15.3)	64.3 (18.3)	68.3 (17.0)	64.0 (20.7)	0.607
	Plasma	51.8 (18.6)	65.9 (19.0)	70.4 (21.8)	67.7 (19.7)	
	Placebo	45.4 (16.2)	62.0 (16.0)	63.3 (15.4)	68.6 (16.7)	
KOOS Sport/Recreation 0-100, mean (SD)	PRP	12.8 (10.9)	27.8 (19.0)	35.5 (21.0)	32.8 (21.1)	**0.031**^d^
	Plasma	29.8 (21.0)	38.8 (27.8)	51.7 (30.3)	46.0 (25.2)	
	Placebo	17.4 (12.2)	33.8 (20.7)	35.7 (16.4)	40.7 (21.6)	
KOOS QOL 0-100, mean (SD)	PRP	18.1 (13.9)	33.1 (21.4)	48.1 (22.0)	39.1 (22.4)	0.336
	Plasma	29.5 (16.4)	42.3 (25.4)	51.5 (27.5)	45.2 (22.7)	
	Placebo	25.0 (15.3)	39.0 (20.2)	43.2 (16.9)	50.6 (22.7)	
OMERACT-OARSI Criteria, %	PRP	-	75%	95%	80%	ns^e^
	Plasma	-	70%	85%	80%	
	Placebo	-	81%	76%	86%	
TUGT (s), mean (SD)	PRP	13.6 (2.8)	13.0 (3.2)	11.6 (1.6)	11.5 (1.3)	0.866
	Plasma	13.8 (3.1)	12.1 (2.7)	12.2 (3.0)	12.2 (3.0)	
	Placebo	13.6 (3.0)	12.3 (2.7)	11.4 (2.0)	11.4 (2.4)	

* This p-value represents difference between groups; there was a statistically significant difference intragroup for all baseline parameters in relation to 6, 12 and 24 weeks

^a^ P-value intergroup week 6: p = 0.823; week 12: p = 0.162; week 24: p = 0.814

^b^ 0 - no improvement; 1 - mild improvement; 2 - moderate improvement; 3 - good improvement; 4 - excellent improvement

^c^ P-value intergroup week 6: p = 0.986; week 12: p = 0.712; week 24: p = 0.076. There was a significant difference in the PRP group between weeks 6 and 12 (p = 0.003) suggesting better responses in week 12

^d^ The difference occurred due to difference between groups PRP and plasma (p = 0.03) from week 6 to week 12 (p = 0.005)

^e^ P-value intergroup week 6: p = 0.767; week 12: p = 0.268; week 24: p = 0.872

*Abbreviations*: *VAS* visual analogue scale 0-10 cm, *SD* standard deviation; ns: no statistical significance, *PhGA* physician global assessment 0-10 cm, *PGA* participant’s global assessment for improvement, *WOMAC* Western Ontario McMaster Universities Osteoarthritis Index, *KOOS* Knee Injury and Osteoarthritis Outcome Score, *ADL* function in daily living, *QOL* quality of life, *TUGT* timed up and go test

For almost all secondary outcomes, there were significant differences between the pre and post-treatment results, with improvement in the parameters evaluated in the 3 groups but no difference between them (Table 2). Participants in the PRP group presented significantly higher frequency of adverse events (65% versus 25% in the plasma group and 33% in the placebo group, p = 0.025) but without difference between groups regarding type, intensity and duration (Table 3). The most common adverse event was pain, of mild or moderate intensity, with a mean duration of 2.1 (SD 1.3) days.

**Table 3:** Summary of all related adverse events

Adverse events, n (%)	PRP (n = 20)	Plasma (n = 21)	Placebo (n = 21)	p-value
No	7 (35%)	16 (76%)	14 (67%)	**0.025**
Yes	13 (65%)	5 (24%)	7 (33%)	
SAEs	0 (0%)	0 (0%)	0 (0%)	-
Deaths	0 (0%)	0 (0%)	0 (0%)	-
TEAEs, n (%)				0.914
Application site pain	8 (40%)	5 (24%)	5 (24%)	
Index knee swelling	1 (5%)	0 (0%)	1 (5%)	
Application site pain and index knee swelling	3 (15%)	0 (0%)	1 (5%)	
Index knee stiffness	1 (5%)	0 (0%)	0 (0%)	
Application site ecchymosis/Bleeding	0 (0%)	0 (0%)	0 (0%)	
Pain intensity, n (%)				0.202
0 - Mild (0 to < 3)	6 (30%)	2 (10%)	4 (19%)	
1 - Moderate (3 to 8)	7 (35%)	2 (10%)	1 (5%)	
2 - Intense (> 8 to 10)	0 (0%)	1 (5%)	2 (10%)	
Duration of pain in days, mean (SD)	2.1 ± 1.3	2.2 ± 1.1	3.6 ± 2.9	0.681

*Abbreviations*: *PRP* platelet-rich plasma, *TEAEs* treatment-emergent adverse events, *SD* standard deviation

It was not possible to perform the analgesic count for each group because few participants adequately completed the diaries, but there was no difference between the groups in a qualitative analysis. For all participants (N = 62), missing data occurred for 6 (9.7%) participants at week 6, 7 (11.3%) at week 12 and 10 (16.1%) at week 24.

Complete post-hoc subanalysis are described in Additional file 2. There was no difference in the VAS for overall pain and function subscale of WOMAC in any of the subgroups analysed such as i) VAS for overall pain <6 versus ≥ 6 cm at baseline and ii) VAS for overall pain comparing two groups (PRP versus placebo; Plasma versus placebo).

## Discussion

The results of this trial showed an improvement in PRP, plasma and placebo groups at weeks 6, 12 and 24 for the main outcomes evaluated: VAS for overall pain, WOMAC, KOOS and OMERACT-OARSI response criteria. However, there was no difference in response between the groups over the time. Treatments were safe with only PRP group showing a higher frequency of mild transitory increase in pain in the days after injections.

A numerical and graphic difference at week 12 favoring PRP group, but without statistical significance, might suggest a beneficial effect of PRP at this timepoint. Similarly, there was a significant statistical difference in the PRP group for the likert scale for improvement from week 6 to 12 (Table 2). However, these finds should be interpreted cautiously given the lack of adjustment for multiple testing. The KOOS subscale sport/recreation also showed different results between groups with the same pattern already identified at baseline.

The current literature is divergent regarding the real effect of PRP for treatment of KOA [12]. This fact may, among other aspects, be due to the lack of standardization of the protocols across the studies, the different techniques for preparation of the PRP, and the different intervals and number of joint injections [12]. The PRP used in this study was fresh, not frozen, obtained by double centrifugation, poor in leukocytes and not activated. It contained an average of 1 x 10^6^ platelets/mm^3^, that is, approximately 3 times the basal platelet count in peripheral blood, applied with a 2-week interval. The advantage of fresh PRP is the theoretical decrease in the loss of function of the platelet products that may occur in the storage of frozen PRP, as was used in some studies [18, 37]. The PRP with low concentration of leucocytes showed previous efficacy for KOA [38] with less local inflammatory reaction [39]. Activation is probably not necessary because it can be achieved through endogenous mechanisms following injection [12] and it is not supported by literature [38]. The double centrifugation might yield higher platelet concentration in PRP [40] but this is also not definitive [38]. Other studies performed a total of 1 to 4 infiltrations, with intervals ranging from 1 to 4 weeks [13, 15, 41]. Ultrasound-guided injections were a differential of our study and, by our knowledge, it was only performed by one previous study [42].

Few studies used placebo as a comparator of PRP so far, all suggesting superiority of PRP [16–20], which differed from the findings of this study. There were methodological issues in these studies such as lack of adequate blinding and use of hyaluronic acid as comparator, which cannot be considered a gold standard for the treatment of OA since there is still a divergence regarding its efficacy [41] and its effect size could have interfered in the quantification of the actual effect size of PRP.

The present study was the first, to our knowledge, to test fresh plasma (not enriched with platelets) as a therapeutic option. The justification was to evaluate whether the possible benefit of PRP demonstrated in other studies was in fact related to platelets and their products or whether there was participation of any other plasma components, such as albumin, coagulation factors, immunoglobulins, electrolytes etc. Autologous conditioned serum, for example, had already been tested for the treatment of KOA due to its anti-inflammatory properties with positive results [43].

The high rate of placebo response in OA treatment trials is a frequent issue that hinders the evaluation of treatments response [44]. It has been reported to occur in around 30% of participants [40], but some studies have reported higher values [45], such as [44] a landmark study of glucosamine and chondroitin for treatment of knee OA, which identified 60% of placebo response [42]. In the present study, the placebo response was around 50% which was likely due to contextual factors related to the close contact of participants with the single researcher who did the enrollment and assessments [46]. Furthermore, it is known that invasive treatments such as injections determine higher placebo responses than oral or topical agents [44].

The improvement in outcomes with saline makes us question whether improvement was due to the placebo effect or whether there is a possible analgesic effect of saline, as has been recently suggested in some reviews and meta-analysis [47–49]. In this regard, saline might have been a confounding factor in several clinical trials in which it was used as a placebo comparator and influenced the divergence of results, although a first trial found no difference between saline and a sham procedure for KOA pain and function in 24 weeks [50]. The mechanism of action of saline could be related to alteration of osmolality in synovial fluid and to a possible participation of sodium in the pathophysiology of OA [47, 51]. Further future studies comparing saline with a sham procedure may help to elucidate this issue.

Adverse effects were twice more frequent in the PRP group compared to the other groups. The most frequent adverse effect reported was mild to moderate increase in knee pain with a mean duration of 2 days. These results were similar to those described by other clinical trials [41].

An important limitation of this study is the relatively small sample, which may have contributed to the absence of difference between the groups. Although we have calculated the sample size using adequate methodology and previously reported SD (1.5 as described by Patel et al [16]), our results showed substantially higher SD (2.5 to 3.3), which may have limited the ability of the study to find differences. In addition, the primary outcome at week 12 might have improved the ability of finding a beneficial effect of PRP in our trial, although previous positive results were showed for 6 to 12 months [16–18].

Other limitations may be pointed out in this study. First, the total volume of injected PRP (1.4 to 5ml) was variable and lower than that used in other studies [15], which might have influenced the PRP efficacy assessment. However, the PRP concentration was the same in all participants and the final absolute platelet count was similar to that injected by Patel et al [16]. It is important to note that there was no guiding standardization for PRP preparation in the literature when the trial was designed [38]. Second, participants were not recommended to washout analgesics before study visits although this is expected to be similar given participants were randomly allocated into one of the study groups. Third, the eligibility criteria were strict in order to assess the effect of PRP and 92% of sample were female, which may limit generalisability. Fourth, we have not performed joint aspiration before the PRP injection in those with joint effusion, although the benefits of this procedure in KOA have not been well established [52, 53]. Finally, we have not done a follow up ultrasonography at the end of the study, which could evaluate the effect of PRP or plasma on structural parameters, such as synovitis, joint effusion and cartilage damage.

## Conclusions

A poor-leucocyte obtained by double centrifugation PRP, injected with a 2-week interval, and plasma were not superior to placebo for pain and function improvement in KOA over 24 weeks, although the relatively small sample may have influenced this finding. The PRP group had a higher frequency of mild transitory increase in pain. Future research comparing PRP to placebo is necessary to confirm the results of this study.

## Supplementary Information

**Additional file 1.**

**Additional file 2.**

## Data Availability

The datasets used and analysed during the current study are available from the corresponding author on reasonable request.

## Abbreviations

BMI: Body-mass index
HA: Hyaluronic acid
KL: Kellgren and Lawrence
KOA: Knee osteoarthritis
KOOS: Knee Injury and Osteoarthritis Outcome Score
PGA: Participant’s global assessment
PhGA: Physician global assessment
PRP: Platelet-rich plasma
SD: Standard deviation
TUGT: Timed up and go test
VAS: Visual analogue scale
WOMAC: Western Ontario McMaster Universities Osteoarthritis Index
