# Supplementary material for: Efficacy of platelet-rich plasma and plasma for symptomatic treatment of knee osteoarthritis: a double-blinded placebo-controlled randomized clinical trial
Source: BMC Musculoskelet Disord. 2021 Sep 24;22:822. doi: 10.1186/s12891-021-04706-7 (PMC8461850; doi:10.1186/s12891-021-04706-7)
Supplement: Supplementary file 1 — Additional file 1. [file 12891_2021_4706_MOESM1_ESM.docx]

**Additional file 1**

The index knee was evaluated by ultrasound with the participant in a supine position with a knee flexion of 30° and maximal flexion (> 90°). The following parameters were evaluated:

1. synovial hypertrophy with gray scale (grades 0 to 4) and presence of power doppler (grade 0 to 4);
2. quantification of joint effusion (measured with knee flexed at 30°, on the longitudinal axis);
3. articular cartilage morphology (grade 0-3):
   1. Grade 0: normal, if they showed a monotonous anechoic band having a sharp hyperechoic anterior and posterior interfaces;
   2. Grade 1: mild degenerative changes: loss of the normal sharpness of cartilage interfaces and/or increased echogenicity of the cartilage;
   3. Grade 2A: moderate degenerative changes, if in addition to above changes, clear local thinning (less than 50%) of the cartilage;
   4. Grade 2B: moderate degenerative changes, if local thinning of the cartilage more than 50% but less than 100%;
   5. Grade 3: severe degenerative changes: 100% local loss of the cartilage.
