# Supplementary material for: Efficacy of platelet-rich plasma and plasma for symptomatic treatment of knee osteoarthritis: a double-blinded placebo-controlled randomized clinical trial
Source: BMC Musculoskelet Disord. 2021 Sep 24;22:822. doi: 10.1186/s12891-021-04706-7 (PMC8461850; doi:10.1186/s12891-021-04706-7)
Supplement: Supplementary file 2 — Additional file 2. [file 12891_2021_4706_MOESM2_ESM.docx]

**Additional file 2**

Subanalysis

Despite the absence of difference between groups, we performed a post-hoc subanalysis to investigate potential predictors of improvement with PRP or plasma treatment:

1. Age ≤ 65 years versus age > 65 years;
2. BMI < 25 versus BMI 25-30 versus BMI > 30 Kg/m^2^;
3. VAS for overall pain <6 versus ≥ 6 cm at baseline;
4. KL2 versus KL3 at baseline;
5. synovitis grades 0 or 1 versus 2 or 3 at baseline;
6. power doppler absent versus present at baseline;
7. joint effusion < 4 mm versus ≥ 4 mm at baseline;
8. VAS for overall pain comparing two groups (PRP versus placebo; Plasma versus placebo);
9. OMERACRT-OARSI "major" responders versus "minor" responders plus non-responders, with “major” responders defined as improvement in pain or function ≥ 50% and absolute improvement ≥ 20;
10. worsening rate (number of participants with worsening in each group);
11. volume of PRP and plasma injected ≤ 3.2 ml versus > 3.2 ml.

There was no difference in the VAS for overall pain and function subscale of WOMAC in any of the subgroups analysed.
